# Gender, Albuminuria and Chronic Kidney Disease Progression in Treated Diabetic Kidney Disease

**DOI:** 10.3390/jcm9061611

**Published:** 2020-05-26

**Authors:** Beatriz Fernandez-Fernandez, Ignacio Mahillo, Jinny Sanchez-Rodriguez, Sol Carriazo, Ana B. Sanz, Maria Dolores Sanchez-Niño, Alberto Ortiz

**Affiliations:** 1 IIS-Fundacion Jimenez Diaz-Universidad Autonoma de Madrid and Fundacion Renal Iñigo Alvarez de Toledo-IRSIN, 28040 Madrid, Spain; beaff26@hotmail.com (B.F.-F.); imahillo@fjd.es (I.M.); jinny.sanchez@quironsalud.es (J.S.-R.); sol.carriazo@quironsalud.es (S.C.); asanz@fjd.es (A.B.S.); mdsanchez@fjd.es (M.D.S.-N.); 2 REDINREN, 28040 Madrid, Spain

**Keywords:** diabetic kidney disease, diabetic nephropathy, gender, outcomes, albuminuria, glomerular filtration rate, mortality

## Abstract

Background: Women are reported to have a lower incidence of renal replacement therapy, despite a higher prevalence of chronic kidney disease (CKD). Aim: To analyze diabetic kidney disease (DKD) progression in men and women. Methods: Prospective cohort: *n* = 261, 35% women, new consecutive nephrology DKD referrals. Results: Women smoked less and better complied with the dietary phosphate and sodium restrictions. Despite a less frequent nephrology referral, women had lower baseline albuminuria. Over a 30 ± 10-month follow-up, albuminuria decreased in women and the estimated glomerular filtration rate (eGFR) loss was slower than in men. However, the percentage of rapid progressors was similar in both sexes. The best multivariate model predicting rapid progression in men (area under curve (AUC) = 0.92) and women differed. Albuminuria and fractional excretion of phosphate (FEphosphate) were part of the men multivariable model, but not of women. The AUC for the prediction of rapid progression by albuminuria was higher in men than in women, and the albuminuria cut-off points also differed. In women, there was a higher percentage of rapid progressors who had baseline physiological albuminuria. Conclusions: Female DKD differs from male DKD: albuminuria was milder and better responsive to therapy, the loss of eGFR was slower and the predictors of rapid progression differed from men: albuminuria was a better predictor in men than in women. Lifestyle factors may contribute to the differences.

## 1. Introduction

There is recent interest in and controversy over the reasons behind the lower incidence of renal replacement therapy (RRT) in women, despite a higher prevalence of chronic kidney disease (CKD) [1,2,3]. The concern that this may represent, that is, decreased access of women to RRT, has been raised [4]. Diabetic kidney disease (DKD) is the most frequent cause of end-stage renal disease (ESRD) and exploring the impact of gender on DKD progression under routine clinical practice conditions may provide information to address the issue [5]. Recent systematic reviews and meta-analyses reported contradictory information on the impact of gender on kidney outcomes in diabetes, ranging from a higher incidence of ESRD in women to no differences in the incidence of albuminuria, GFR decline or ESRD to a higher incidence of albuminuria or ESRD in men [6,7]. The apparent contradictory data regarding the impact of gender on DKD may be ascribed to sex hormones, to differences in lifestyles between different countries or to differences in access to healthcare between countries, including potential differences between genders in access to healthcare. In this regard, Spain has a free at point-of-care public healthcare system that precludes potential differences in access to care and is projected to become the country with the longest life expectancy by 2040 [8]. Additionally, there is no restriction in access to specialist care. In a global context of increasing life expectancy and progressively increased access of the population to healthcare, Spain may represent a model system in which to understand current and global trends in DKD. Indeed, DKD is the most frequent cause of CKD in Spain and CKD is projected to become the second most common cause of death before the end of the century, thus tracking global trends in the increasing CKD impact on mortality [8,9]. Understanding what the drivers are of DKD in different populations, including differences between genders, is key to develop effective preventive and therapeutic strategies.

We have now explored gender differences in DKD characteristics and progression in a contemporary cohort treated at an outpatient nephrology clinic following clinical guidelines.

## 2. Patients and Methods

This is an observational, prospective study of 261 incident patients with CKD and diabetes mellitus (DM) in a monographic outpatient nephrology clinic of a tertiary hospital. Most patients had type 2 diabetes, but patients with type 1 diabetes (9 (5.3%) men and 5 (5.5%) women) were not excluded so as to more closely reflect the routine clinical practice. Patients were spontaneously referred for nephrological evaluation by primary care physicians or other specialists and referred patients were consecutively enrolled. The protocol was approved by the IIS-Fundacion Jimenez Diaz Ethics Committee (EO030-20). Participants signed an informed consent form before using their information for this research. Inclusion criteria were adults with type 1 or type 2 DM, referred to nephrology between March 2011 and September 2014 because of pathological albuminuria (urinary albumin/creatinine ratio, UACR > 30 mg/g) or a pathological estimated glomerular filtration rate (eGFR < 60 mL/min/1.73 m^2^) that did not have evidence for another cause of CKD. Exclusion criteria were being less than 18-years-old, on RRT, having positive serology for hepatitis B virus (HBV), hepatitis C virus (HCV) or human immunodeficiency virus (HIV) or unwilling to participate. DKD diagnosis was clinical. Medication was prescribed according to ADA and KDIGO guidelines for DKD prior to the introduction of the 2019 recommendations of sodium glucose cotransporter-2 (SGLT2) inhibitors for DKD [10,11]. Patients were followed until the initiation of RRT, death or 30 October 2015 for a mean follow-up of 30 ± 10 months.

The CKD-EPI formula was used for the eGFR calculation [12]. CKD albuminuria (A) and GFR (G) categories were defined according to KDIGO [11]. All patients had a complete clinical history, including an assessment of treatments, blood and urine tests, electrocardiogram and transthoracic echocardiogram. Hypertension was defined as office systolic blood pressure >140 mmHg or diastolic blood pressure > 90 mmHg or the intake of blood pressure lowering drugs for hypertension. Cardiovascular disease (CVD) was defined as congestive heart failure, acute coronary syndrome including myocardial infarction with an ST segment elevation (representing the isoelectric period between depolarization and repolarization of ventricles, that rises up in acute coronary injury) in the electrocardiogram, myocardial infarction without an ST elevation and unstable angina, cardiac arrhythmia as defined by an electrocardiogram (chronic or paroxysmal atrial fibrillation, atrial flutter, and atrioventricular blocks), intermittent claudication or peripheral vascular disease, or stroke diagnosed using computed tomography [13].

Outcomes were defined as follows: eGFR slope was calculated in ml/min/1.73 m^2^/year as (Final eGFR-initial eGFR)/follow-up time (years). Percentage change in UACR was calculated as ((final UACR-initial UACR)/initial UACR) × 100)/follow-up time (years). Patients were followed until death or the initiation of RRT.

### Statistical Analysis

Continuous variables are described as mean ± standard deviation (SD) for normal distributions or median (interquartile range (IQR)) for non-normal distributions, including a 95% confidence interval (CI) when appropriate. Means were compared by parametric tests (Student’s t test for 2 groups and ANOVA for 3 or more groups), and medians by non-parametric tests (Mann–Whitney U or Kruskal–Wallis). A Pearson correlation coefficient or Spearman Rho were used for assessing the correlation between the continuous variables. The evaluation of the association between the categorical variables was performed using an χ^2^ test or Fisher test when the frequency was low (5 or less individuals). For the quantification of the associations, multivariate linear regression models or logistic regression were applied, according with the nature of the variable (dependent, continuous or binary). Variables were compared with ANOVA for repeated measures, and UACR was log transformed. To study the variables associated with the loss of renal function, the eGFR slope was the dependent variable and other baseline variables were considered independent variables (or prognostic factors). Additionally, progression was categorized as rapid progressors (loss of eGFR > 5 mL/min/1.73 m^2^/year) or standard progressors (loss of < 5 mL/min/1.73 m^2^/year), following KDIGO 2012 [11]. The odds ratio (OR) was determined with logistic regression models. For assessing the variables associated with UACR progression, UACR was categorized into tertiles, comparing the highest and lowest tertiles. In logistic regression models, the OR was presented as a measure of risk, and a multivariate analysis was performed using the significant variables (*p* < 0.05) or *p* < 0.1, according to the univariate analysis adjusted by age, eGFR (CKD-EPI equation) and baseline UACR. Receiver operating characteristic (ROC) curves were used to graphically represent the best model. Multivariate models were constructed stepwise, using as candidate variables those that in the univariate analysis presented *p* < 0.1. Inclusion or exclusion of variables in the stepwise model was based on the likelihood ratio. All studies were performed for the total population, and for men and women separately. A total of 18 (7%) patients were lost to follow-up: 9 (5%) men and 9 (9%) women.

## 3. Results

### 3.1. Clinical and Analytical Characteristics of Men and Women

261 incident patients were referred to the DKD nephrology clinic and accepted to participate in the study cohort. Two thirds of the cohort (65%) were men and one third (35%) were women (Table 1). There were no significant differences between men and women in age, eGFR, diabetic complications, cardiovascular disease or cardiovascular risk factors such as hypertension or dyslipidemia (Table 1). However, women had lower baseline albuminuria, assessed as either 24 h albuminuria or UACR and were more frequently non-smokers (Table 1). In this regard, the distribution in the GFR G categories was similar in men and women (Figure S1A) but in men, the albuminuria A categories were skewed towards A3 as compared with women (Figure S1B). Peripheral artery disease was also less frequent in women. Women had a higher BMI and lower waist circumference. However, according to the 2013 AHA/ACC/TOS overweight and obesity guidelines, the cardiometabolic risk is high if the waist circumference is >102 cm for men and >88 cm for women and, in this regard, the waist circumference in women was higher above these limits than in men.

There were no significant differences in the use of medications with known antiproteinuric properties such as renin–angiotensin system (RAS) blockers, anti-aldosterone agents, diuretics, pentoxifylline or statins (Table S1) and no patient was on SGLT2 inhibitors.

Among the key laboratory parameters, women had lower hemoglobin, transferrin saturation, ferritin and 25OH vitamin D levels and higher folic acid, HDL cholesterol and alkaline phosphatase (Table S2). By contrast, the parameters of glycemia control and inflammation did not differ. There were some differences in the 24-h urine excretion of electrolytes, likely reflecting differences in diet. Thus, women excreted 17% less sodium, 22% less phosphate, 21% less magnesium and 15% less potassium in 24 h than men, despite similar serum levels of these parameters (Table S2).

### 3.2. GFR Loss in Men and Women

The overall outcome was good. Albuminuria remained stable in men changing by 3.1 [−7.3; 20.3] %/year (*p* = 0.74), but decreased in women, changing by −4.8 [−12.9; 7.4] %/year (*p* = 0.042) during a mean follow-up of 30 ± 10 months. The loss of eGFR was within the range described for the general population. At the end of the follow-up, eGFR had changed by −1.2 [−4.6; 2.3] ml/min/1.73 m²/year in men (*p* = 0.011) and −0.8 [4.1; 3.5] ml/min/1.73 m²/year in women (*p* = 0.07). However, 48/261 (18%) patients showed rapid progression, defined as a loss of ≥5 mL/min/1.73 m²/year over the follow-up period [11]. Women represented 15/48 (31%) of rapid progressors and 70/199 (35%) of non-rapid progressors.

The univariate predictors of rapid progression were adjusted for age, eGFR and UACR. The HbA1C values were not significantly associated with rapid progression (OR 95% CI 0.959; 0.735 to 1.237, *p* = 0.751). Some variables were associated with rapid progression both in men and in women such as lower 25OH vitamin D levels and higher UACR (Table 2). However, most of the statistically significant predictors differed for men and women.

The best multivariate model to predict rapid progression in the whole cohort included UACR, FEphosphate, triglycerides, uric acid and vitamin B12 (Table 3). The area under the ROC curve (AUC) to predict rapid progression was 0.81 (95% CI 0.73–0.89) (Figure S2a). However, the best multivariate model to predict rapid progression in men and women differed. UACR and FEphosphate were part of the multivariable model predicting rapid progression in men (Table 3), which had an AUC of 0.92 (95% CI 0.84–1.00) (Figure S2b). When applied to women, this model had an AUC of 0.76 (not shown). By contrast, UACR and FEphosphate were absent from the women model, while folic acid, systolic blood pressure (SBP) and uric acid were present (Table 3). The women model had an AUC of 0.90 (95% CI 0.80–1.00) to predict rapid progression (Figure S2c). When applied to men, this multivariate model resulted in an area under the ROC curve of 0.67.

Interestingly when UACR was added to the multivariable model in women, it was not statistically significant, but then folic acid also became not statistically significant (Table S3a). In this new model, the removal of folic acid resulted in UACR becoming significant (Table S3b). This suggests that folic acid and UACR may be providing information on a shared pathway.

### 3.3. Predictive Value of Baseline UACR for Rapid Progression

**Figure 1:**
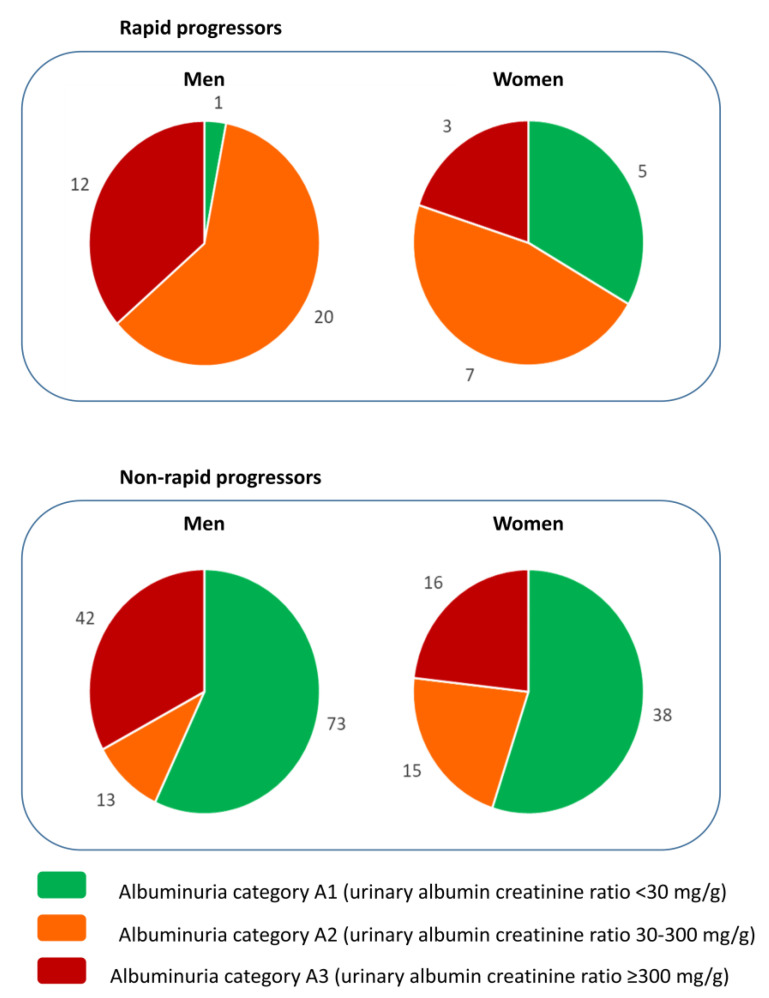
Baseline UACR A category and rapid progression according to sex. The number of patients in each albuminuria A category shown separately for men and for women among those that had a rapid loss of GFR (defined as loss of eGFR >5 mL/min/1.73 m^2^/year according to KDIGO 2012 [11]) and those without a rapid loss of eGFR. Albuminuria A category defined as per KDIGO 2012 [11]. Note the presence of more women than men with rapid progression in the A1 category representing the physiological albuminuria values.

**Figure 2:**
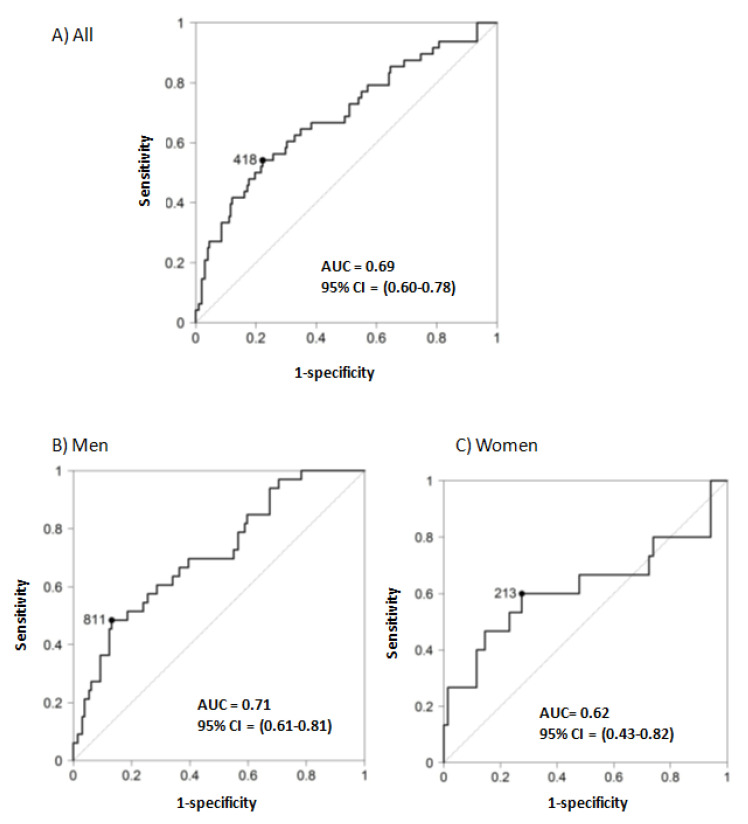
Receiver operating characteristic (ROC) for baseline UACR as a predictor of rapid progression. Area under the curve (AUC) of the receiver operating characteristic (ROC) for the prediction of rapid progression (defined as loss of eGFR > 5 mL/min/1.73 m^2^/year according to KDIGO 2012 [11] by baseline urinary albumin/creatinine ratio (UACR). (**A**) All patients. (**B**) Men. (**C**) Women, CI: confidence interval.

The thresholds that define A2 and A3 albuminuria are based on the risk of CKD progression and, accordingly, baseline UACR was associated with the rapid loss of GFR in the whole cohort, as well as in men and in women separately (Table 2 and Table 3). However, a significant percentage of patients with rapid progression had UACR < 30 mg/g (12.5% of patients) or <300 mg/g (69% of patients), and 30% of patients with baseline UACR > 300 mg/g did not have rapid progression. The optimization of renal care during the follow-up in the DKD nephrology clinic may have contributed to this later observation. However, the most striking observation is that in women, the percentage of rapid progressors with baseline UACR < 30 mg/g (5/15, 33%) was higher than in men (1/33, 3%; *p* = 0.0085, Fisher exact test) (Figure 1). Indeed, the AUC of the ROC for the prediction of rapid progression by baseline UACR was 0.69 (95% CI 0.60–0.78) for the whole population, 0.71 (95% CI 0.61–0.81) for men and 0.62 (95% CI 0.43–0.82) for women, with UACR cut-off points of 418, 811 and 213 mg/g, respectively (Figure 2). Of note, the 95% CI of the female AUC included 0.50, indicating that UACR was not a good predictor of rapid GFR loss in women.

## 4. Discussion

We have presented data on DKD nephrology care and outcomes in Spain, the country projected to have the longest life expectancy by 2040 [8]. This specific position of Spain in global epidemiology may make this information of interest for other healthcare systems. The main finding is that the DKD presentation and outcomes differ for males and females. These differences are paralleled by differences in lifestyle factors known to or suggested to influence albuminuria and CKD progression, including smoking behaviors and compliance with low-sodium, low-phosphate and low-potassium diets. More specifically, baseline albuminuria was lower in females despite a lower number of female referrals and similar baseline GFR, hypertension, diabetes control and diabetes complications between sexes. Additionally, albuminuria further decreased during the follow-up only in females, pointing to major differences in the clinical presentation of DKD between men and women. Thus, females, as a group, did not significantly lose eGFR during the follow-up period and, although a subset of them were rapid progressors, the multivariate model to best predict rapid progression differed between males and females and albuminuria was not a good biomarker of rapid progression in women. This calls for the development of female-specific DKD progression risk equations that help enrich clinical trials in women at high risk of DKD progression.

The first interesting observation relates to the different lifestyles that may have contributed to the differences between males and females, as the mean women age was 70 years, and most were post-menopausal (85% of them had >55 years), so the impact of female hormones would be expected to be low. Thus, women with DKD were less frequently smokers and ingested (as assessed by 24 h urinary excretions) less sodium, less phosphate and less potassium. Smoking has been associated with incident albuminuria in the general population and with albuminuria in systematic reviews of type 2 DM [14,15]. Lower sodium ingestion is associated with lower albuminuria and in clinical trials, lowering sodium ingestion decreased albuminuria in DKD [16]. Specifically, the dietary sodium restriction increased the anti-albuminuric effect of RAS blockade, which is relevant for our study [16]. In this regard, a lower potassium intake may facilitate achieving an optimal dosing of RAS blockers [17]. Finally, a bidirectional relationship between the albuminuria and phosphate balance has been described. Thus, patients with proteinuria may have a more limited capacity to excrete a phosphate load, given the existence of renal resistance to the phosphaturic hormone FGF-23, likely driven by albuminuria-induced kidney depletion of the FGF-23 co-receptor Klotho [18,19,20]. On the other hand, high phosphate levels are associated with suboptimal kidney protection by RAS blockade [21].

The present cohort, based on the routine clinical practice referral of patients to nephrology, was composed of a majority of men. Despite the lower number of females and the similar eGFR, women had lower baseline albuminuria values. The observation is in line with recruitment data from recent trials. For example, in the CANVAS trial testing add-on canagliflozin or placebo for DKD patients with UACR 300 mg/g despite RAS blockade, just 34% of patients were women, likely reflecting the lower overall albuminuria values of women while on RAS blockade [22,23]. Overall, it points to a better response of females to RAS blockade, which may be favored by the lifestyle differences pointed out above, or to lower albuminuria values even before RAS blockade. Our data of a further decrease in albuminuria during the nephrological follow-up in females but not in males supports the notion of better response to therapy. Although compliance with prescribed medication could not be formally assessed in routine clinical practice, it could be a contributor, given the better female compliance with smoking and dietary advice. These behavioral differences between genders may differ in different countries, especially at the age range of the studied population. Thus, female uptake of smoking had not yet become as popular for thisgeneration of Spanish women as in other developed countries. In any case, during the follow-up, blood pressure was well controlled in the full population, using nephroprotective treatments with a relatively low total sodium intake, especially in women. In these conditions, the CKD progression slope assessed by eGFR was like that reported in the general population (−1 mL/min/1.73 m^2^/year), in accordance with recent reports in DKD [24]. This is in line with prior reports from Spain: in a retrospective cohort of 197 diabetic kidney disease patients, the loss of eGFR in the first year of follow-up was −2.43 ± 7.88, −1.96 ± 9.3 and −0.15 ± 10.66 mL/min/1.73 m^2^ for those not on, intermittently on or constantly on RAS blockade for an overall annual eGFR loss of eGFR of−1.2 ± 9.6 mL/min/1.73 m^2^, although no gender-specific data were provided [25]. However, there was a 20% of male and 18% of female population that were considered as rapid progressors by using the standard definition of the loss of eGFR >5 mL/min/1.73 m^2^/year [11]. This residual risk is the current battleground in DKD and there is evidence that still some patients progress despite the recent availability of SGLT2 inhibitors [23]. The identification in advance of these patients at risk of rapid GFR loss is key to enrich future clinical trials in high-risk patients. In this regard, SGLT2 inhibitors decreased albuminuria and were kidney protective in RCTs in DKD patients, but whether there is a differential impact in women and men remains unclear [23]. In this regard, for the whole population and for males treated with RAS blockade, our data confirmed that albuminuria is a key risk factor and likely a primary driver of DKD progression. However, the predictive value of albuminuria was lower in females. Thus, in females, the AUC for the ROC of the rapid progression prediction by albuminuria was no different from tossing a coin. Furthermore, multivariate models for predicting rapid progression differed in men and women, with albuminuria absent from the best female multivariate predictor of rapid progression. In this regard, while the baseline UACR cut-off point for the ROC to predict rapid progression was very similar in the whole population (418 mg/g) to the cut-off point used to define A2 albuminuria (300 mg/g), this overall cut-off point resulted from very different cut-off points for men (811 mg/g) and women (213 mg/g). Although there have been conflicting data, an individual level meta-analysis, using pooled data from the 2,051,158 general population of high risk and CKD participants, further supports the development of different cut-off points for UACR for men and women [26]. Thus, the slope of the risk relationship of UACR for all-cause and cardiovascular mortality was steeper in women than in men, despite the risk being higher in men at all levels of UACR. Interestingly, for UACR between 15 and 350 mg/g, women showed a steeper risk relationship with ESRD than men: compared with a UACR of 100 mg/g, the hazard ratio associated with UACR 300 mg/g was 1.63 (1.33 to 1.99) in women and 1.33 (1.04 to 1.69) in men (*p* for interaction <0.01) [26]. The steeper slopes in women for both death and ESRD, especially at lower UACR ranges, are in line with our data identifying a lower UACR level as associated with risk in women than in men, despite the meta-analysis not observing overall differences between men and women over the whole range of the UACR values with respect to ESRD. The lower amounts of urine creatinine in women than in men may further compound the problem.

In our population, higher values of baseline UACR and lower eGFR, either raw or adjusted by baseline eGFR, UACR and age, were associated with rapid progression, so too were classical risk factors as systolic hypertension and hypertriglyceridemia. Both sexes shared higher baseline UACR and lower 25OH Vitamin D as common factors for rapid progression, while other factors differed. It is of interest that five out of ten variables that differed in the best multivariate models for males and females corresponded to variables that were statistically different between sexes at baseline (*p* < 0.05) or that approached statistically significant differences (*p* < 0.1). At this stage, we cannot differentiate between different sensitivity of females and males to different biological variables from the existence of a different range of these values between males and females, that allows to observe differences in the impact of outcomes only for the range of values corresponding to one of the sexes. Interestingly all the components in both multivariable models are potentially modifiable by therapy.

In women, but not in men, lower folic acid levels were a risk factor for rapid progression. Differences between sexes in baseline folate levels may have impacted our ability to discriminate an impact of folate in men. Our data are in line with the longstanding but conflicting reports on the potential association of genetic variants of methylenetetrahydrofolate reductase [27,28,29,30], hyperhomocysteinemia and/or folate and vitamin B12 levels with CKD progression in different contexts, including diabetes [28,29,31,32,33,34,35,36]. In this regard, in a recently published substudy of the China Stroke Primary Prevention Trial (CSPTT) in hypertensive adults, the combination of 10 mg enalapril and 0.8 mg folic acid was associated with a slower CKD progression than enalapril alone [37]. This study may suggest the need for baseline RAS blockade to observe the benefits of folate supplementation. In this regard, in our study linking folate levels to DKD outcomes, most patients were on RAS blockade. Additionally, the CSPTT authors remarked that the protective result obtained may be explained by the lower baseline folate levels (7.7 ng/mL) than in prior negative trials of vitamin supplementation (mean baseline folate levels 15 to 16 ng/mL). In this regard, the baseline folate levels in our study population were in the range (6.65–7.86 ng/mL) observed in the CSPTT trial.

Several limitations should be acknowledged. As in most epidemiological studies, eGFR was used to assess renal function and the conclusion might have differed if GFR had been measured. Additionally, the sample size was smaller than other large epidemiological studies. However, the smaller sample size allowed to collect information and urinary variables that cannot be usually collected in these larger epidemiological studies. Being an observational study, it did not generate information on causality. Despite RCTs pointing to a link between folate and CKD progression, the biological basis linking folate levels to kidney outcomes are unclear. As a further limitation, income data were not available, precluding the analysis of the impact of socioeconomic class. Additionally, no formal compliance assessment tool was used. Regarding the variables exploring tubular function, such as the fractional excretion of solutes, the observational nature of the study precludes addressing the pathophysiological factors that modify tubular function variables and are responsible for their association with outcomes. Experimental and interventional studies will be required to address the pathophysiology behind the observations. Finally, the study was performed in the pre-SGLT2 inhibitor era. Thus, conclusions may not apply to patients on SGLT2 inhibitors. However, most of the world population is not expected to have widespread access to these more expensive medications in the near future.

## 5. Conclusions

In conclusion, DKD in the era of RAS blockade appears to differ between males and females, as females have lower albuminuria and better albuminuria responses and overall rates of GFR loss. To what extent better compliance with healthy lifestyles contributes to these differences should be further addressed in other societies with less lifestyle differences between men and women. Under these circumstances, albuminuria is a worse predictor of outcomes in females than in males. Future studies should reconsider the cut-off points for the definition of albuminuria as a predictor of high risk for rapid progression in diabetic women and men. It is worth exploring alternative definitions of high risk in females, encompassing alternative variables. In this regard, the multivariate models identified in the present analysis for the prediction of rapid progression in females are mostly composed of modifiable variables.

## Figures and Tables

**Table 1:** General baseline patient characteristics. Data presented as mean ± SD, median (IQR) or n (%).

Variable	Total (*n* = 261)	Men (*n* = 170)	Women (*n* = 91)	*p*
Age (years)	68 ± 13	68 ± 12	70 ± 13	0.13
Ethnicity, *n* (%)				
Caucasian	243 (93.1)	156 (91.7)	87 (95.0)	0.84
Black	9 (3.4)	6 (3.5)	3 (3.3)	
Asiatic	9 (3.4)	8 (4.5)	1 (1.1)	
Serum creatinine (mg/dL)	1.50 ± 0.80	1.58 ± 0.83	1.36 ± 0.71	0.003
eGFR CKD-EPI (mL/min/1.73 m^2^)	54.8 ± 25.8	56.3 ± 25.7	52.0 ± 25.8	0.20
Urinary creatinine (mg/24h)	1120 ± 398	1253 ± 395	858 ± 245	<0.0001
Albuminuria (mg/24 h)	182 [41–515]	247 [60–645]	87 [12–275.1]	0.0002
UACR (mg/g)	156 [37–499]	187 [50–592]	99.5 [15–403]	0.013
HbA1C (%)	7.6 ± 1.36	7.63 ± 1.32	7.57 ± 1.4	0.71
Diabetes complications, *n* (%)				
Diabetic retinopathy	127/261 (49.7)	81/170 (47.6)	46/91 (50.5)	0.19
Mild/moderate/non-proliferative	72/261 (27.6)	50/170 (29.4)	22/91 (24.2)	
Severe of proliferative	33/261 (12.6)	16/170 (9.4)	17/91 (18.7)	
Macular edema	22/261 (8.4)	15/170 (8.9)	7/91 (7.7)	
Diabetic polyneuropathy	34/261 (13.0)	22/170 (12.9)	12/91 (13.2)	1
Cardiovascular disease, *n* (%)	128/261 (49.0)	83/170 (48.8)	45/91 (49.5)	1
Heart failure	42/261 (16.1)	31/170 (18.2)	11/91 (12.0)	0.26
Acute coronary syndrome	61/261 (23.4)	42/170 (24.7)	19/91 (20.9)	0.58
Stroke	16/261 (6.1)	8/170 (4.7)	8/91 (8.8)	0.29
Peripheral vascular disease	62/261 (23.8)	48/170 (28.2)	14/91 (15.4)	0.03
Arrhythmia	38/261 (14.6)	20/170 (11.8)	18/91 (19.8)	0.11
Tobacco use, *n* (%)				
Non-smoker	108/261 (41.4)	37/170 (21.9)	71/91 (78.0)	<0.001
Smoker	49/261 (18.8)	39/170 (23.1)	10/91 (11.0)	
Ex-smoker	103/261 (39.5)	93/170 (55.0)	10/91 (11.0)	
Hypertension, *n* (%)	251/261 (96.2)	165/170 (97.1)	86/91 (94.5)	0.19
Dyslipidemia, *n* (%)	232/261 (88.9)	149/170 (87.7)	83/91 (91.5)	0.62
SBP (mmHg)	137.7 ± 17.8	136.8 ± 15.8	139.5 ± 21.1	0.29
DBP (mmHg)	73.5 ± 12.6	74.6 ± 13.1	71.6 ± 10.8	0.052
BMI (kg/m²) (*n* = 260)	30.2 ± 5.3	29.5 ± 5	31.3 ± 5.7	0.0073
Waist circumference (cm) (*n* = 180)	107 ± 13	108 ± 13	103 ± 12	0.046

UACR: urinary/albumin creatinine ratio; BMI: body mass index, DBP: diastolic blood pressure; SBP: systolic blood pressure.

**Table 2:** Variables associated with rapid progression in men and women. Variable shown if *p* value ≤0.05, adjusted by eGFR measured by the Chronic Kidney Disease Epidemiology Collaboration) equation (CKD-EPI), age and urinary albumin/creatinine ratio (UACR) are shown.

**Men**	**Adjusted Models**		
	**OR**	**95% CI**	***p***
eGFR CKD-EPI (ml/min/1.73 m^2^)	1.024	(1.005–1.044)	0.011
Alkaline phosphatase (UI/mL)	1.017	(1.003–1.031)	0.016
Triglycerides (mg/dL)	1.007	(1.001–1.013)	0.019
Vitamin B12 (pg/mL)	1.003	(1.000–1.006)	0.024
Albumin (g/dl)	0.196	(0.051–0.710)	0.013
Diuresis (ml/24h)	0.999	(0.998–1.000)	0.026
Waist circumference (cm)	0.944	(0.900–0.986)	0.008
BMI (kg/m²)	0.907	(0.821–0.991)	0.03
FECa (%)	0.364	(0.128–0.860)	0.019
FE phosphate (%)	0.891	(0.818–0.958)	0.001
25 OH Vitamin D (ng/mL)	0.941	(0.888–0.991)	0.021
UACR (mg/g)	1.001	(1.001–1.002)	0.000
**Women**	**Adjusted models**		
	**OR**	**95% CI**	***p***
Leucocytes (*n*/µL)	1.000	(1.000–1.001)	0.032
Age (years)	1.085	(1.012–1.185)	0.019
hsCRP (mg/dL)	3.806	(1.368–22.177)	0.004
SBP (mmHg)	1.040	(1.007–1.081)	0.016
Heart rate (bpm)	1.067	(1.002–1.144)	0.044
Folic acid (pg/mL)	0.712	(0.520–0.893)	0.001
FE potassium (%)	0.782	(1.099–1.563)	0.001
FE magnesium (%)	0.661	(0.420–0.966)	0.031
25 OH Vitamin D (ng/mL)	0.886	(0.782–0.970)	0.006
UACR (mg/g)	1.001	(1.000–1.002)	0.008

UACR: urinary/albumin creatinine ratio; BMI: body mass index, SBP: systolic blood pressure, FE: fractional excretion; hsCRP: high sensitivity C reactive protein; CI: confidence interval.

**Table 3:** Multivariate model for the prediction of rapid progression in the total population (men and women combined), men and women.

**Total Population**				
**Variable**	**Coef.**	**OR**	**95% CI**	***p*** ****
UACR (mg/g)	0.002	1.002	(1.001–1.003)	0.000
FE phosphate (%)	−0.111	0.895	(0.841–0.944)	0.000
Triglycerides (mg/dL)	0.006	1.006	(1.002–1.011)	0.006
Uric acid (mg/dL)	0.327	1.387	(1.080–1.800)	0.010
Vitamin B12 (pg/mL)	0.003	1.003	(1.001–1.006)	0.001
Constant	−4.561			
**Men**				
**Variable**	**Coef.**	**OR**	**95% CI**	***p***
UACR (mg/g)	0.002	1.002	(1.001–1.004)	<0.001
FE phosphates (%)	−0.184	0.832	(0.719–0.920)	<0.001
Waist circumference (cm)	−0.082	0.921	(0.848–0.986)	0.015
Vitamin B12 (pg/mL)	0.006	1.006	(1.002–1.011)	0.005
Mean corpuscular volume (fl)	0.210	1.234	(1.032–1.582)	0.018
DDLV (mm)	0.120	1.127	(1.001–1.291)	0.047
Constant	−17.44			
**Women**				
**Variable**	**Coef.**	**OR**	**95% CI**	***p***
Folic acid (pg/mL)	−0.250	0.779	(0.555–0.978)	0.029
SBP (mmHg)	0.075	1.078	(1.029–1.150)	<0.001
FE Mg (%)	−0.447	0.639	(0.391–0.939)	0.020
Uric acid (mg/dL)	0.620	1.859	(1.103–3.503)	0.019
Constant	−12.91			

UACR: urinary/albumin creatinine ratio; FE: fractional excretion; DDLV: diastolic diameter left ventricle, SBP: systolic blood pressure, FE: fractional excretion.

## Supplementary Materials

The following are available online at https://www.mdpi.com/2077-0383/9/6/1611/s1, Figure S1: Distribution of men and women in CKD eGFR G and albuminuria A categories at baseline. (A) Percentage of men and women per CKD eGFR G category. (B) Percentage of men and women per CKD albuminuria A category. G and A categories defined as per KDIGO 2012, Figure S2. ROC for best baseline multivariable model predictor of rapid progression. Area under the curve (AUC) of the receiver operating characteristic (ROC) for prediction of rapid progression (defined as loss of eGFR >5 ml/min/1.73 m^2^/year according to KDIGO 2012 by the best baseline multivariable model. (A) All patients. (B) Men. (C) Women, Table S1: Baseline medications, Table S2: Key baseline laboratory values, Table S3A and B. Multivariate model for prediction of rapid progression in women, adding UACR to the best multivariate model.
