# Supplementary material for: Gender, Albuminuria and Chronic Kidney Disease Progression in Treated Diabetic Kidney Disease
Source: J Clin Med. 2020 May 26;9(6):1611. doi: 10.3390/jcm9061611 (PMC7356286; doi:10.3390/jcm9061611)
Supplement: Supplementary file 1 [file jcm-09-01611-s001.pdf]

Journal of
Clinical Medicine

MDPI

Supplementary materials

Gender, albuminuria and chronic kidney disease progression in treated diabetic kidney disease

Beatriz Fernandez-Fernandez[1,2], Ignacio Mahillo[1], Jinny Sanchez-Rodriguez[1,2], Sol Carriazo[1,2], Ana B Sanz[1,2], Maria Dolores Sanchez-Niño[1,2] and Alberto Ortiz[1,2*]

[1] IIS-Fundacion Jimenez Diaz-Universidad Autonoma de Madrid and Fundacion Renal Iñigo Alvarez de Toledo-IRSIN, Madrid, Spain
[2] REDINREN, Madrid, Spain
* Correspondence: aortiz@fjd.es; Tel.: +91-5550-48-00

Table S1. Baseline medications; n (%).

Medication	Total n=261	Men n=170	Women n=91	P
RAS blockade	246 (94.3)	162 (95.3)	84 (92.3)	0.48
ACE inhibitor	107(40.6)	72 (42.4)	35 (38.5)	0.63
ARB	170(65.1)	115 (67.6)	55 (60.4)	0.33
Anti-aldosterone	15 (5.7)	11 (6.5)	4 (4.4)	0.68
Diuretics	160 (61.3)	105 (61.8)	55 (60.4)	0.94
Statins	217 (83.1)	140 (82.4)	77 (84.6)	0.77
Pentoxifylline	4 (1.5)	3 (1.8)	1 (1.1)	1
Any vitamin D	101 (38.7)	67 (39.4)	34 (37.4)	0.74
Phosphate binders	32 (12.3)	18 (10.6)	14 (15.4)	0.35

RAS: renin-angiotensin syndrome; ACE angiotensin converting enzyme; ARB: angiotensin receptor blocker.

Table S2. Key baseline laboratory values.

Laboratory value	n	Total	Men	Women	p
Leucocytes (10^3 /µl)	261	8302±2720	8283±2244	8297±3497	0.56
Hemoglobin (g/dl)	261	13.37±1.73	13.79±1.69	12.57±1.52	**0.000**
Mean corpuscular volume (fl)	261	90.4 [87.3-93.9]	90.5 [87.9-93.4]	90 [85.7-93.4]	0.23
Transferrin saturation (%)	257	22 [17-29]	24 [18-30]	20 [18-26]	**0.003**
Ferritin (ng/ml)	259	84 [48.5-178.5]	103 [53-188.5]	70 [32.5-140.2]	**0.009**
Vitamin B12 (pg/ml)	234	412 [307-560]	400 [307-511]	431 [295-572]	0.5
Folic Acid (pg/ml)	229	7.11 [4.78-10.3]	6.65 [4.73-9.47]	7.86 [5.13-11.66]	**0.024**
Glucose (mg/dl)	261	143±54	132 [109-166]	133 [101-173]	0.90
HbA1C (%)	260	7.6±1.36	7.63±1.32	7.57±1.4	0.71
Uric acid (mg/dl)	260	6.58±1.67	6.72±1.54	6.334±1.88	0.056
Albumin (g/dl)	259	4.11±0.38	4.2±0.6	4.1±0.4	0.49
Total Cholesterol (mg/dl)	261	156.8±38.4	152.9±36.6	155±39.5	**0.017**
LDL Cholesterol (mg/dl)	256	85±31	81±32.5	79±34	0.86
HDL Cholesterol (mg/dl)	256	45±13	40±12	48±19	**0.000**
Triglycerides (mg/dl)	261	142±103	121.5±73	121±68	0.81
CRP (mg/dl)	149	0.28 [0.11-0.75]	0.25[0.07-0.66]	0.35[0.14-1.1]	0.21
Sodium (mmol/L)	260	139.59±2.98	139.5±3.05	139.7±2.8	0.65
Potassium (mmol/L)	260	4.65±0.47	4.65±0.46	4.64±0.47	0.76
Total CO_2 (mmol/l)	229	26.90±3.88	27.04±3.95	26.65±3.75	0.38
Diuresis (mL)	244	1855.7±592.6	1918.2±628.8	1732.3±494.6	**0.022**
Urinary Sodium (mmol/24h)	228	154±69	164.3±71.4	136.8±62.5	**0.0037**
Urinary Potassium (mmol/24h)	228	61.58±23.38	64.82±24.55	55.23±19.54	**0.0035**
Serum calcium corrected (mg/dl)	260	9.39±0.43	9.37±0.41	9.43±0.47	0.27
Serum phosphate (mg/dl)	258	3.64±0.56	3.60±0.58	3.72±0.53	0.39
PTH (pg/ml)	254	57.5 [39.6-92.6]	56.7 [35.2-92.15]	59.5 [42.35-98.9]	0.18
Serum magnesium (mg/dl)	239	1.9±0.43	1.92±0.27	1.88±0.25	0.29
1.25 $(OH)_2$ Vitamin D (pg/mL)	141	30.1±11.1	30.2±11.4	30.1±10.4	0.99
25 OH Vitamin D (ng/ml)	255	18.2±10.6	19.08±9.24	16.53±12.70	**0.0002**
Alkaline phosphatase (UI/l)	260	88.1±41.0	83.28±29.98	97.17±55.83	**0.0045**
Urinary calcium (mg/24h)	221	48.15 [27.22-107.95]	48.37 [28-111.12]	48.9 [25.5-98.1]	0.56
FE calcium (%)	232	0.77 [0.37-1.19]	0.73 [0.38-1.18]	0.88 [0.36-1.3]	0.28
Urinary phosphate (mg/24h)	221	621.09±252.81	670.02±264.49	521.89±193.84	**<0.0001**
FE phosphate (%)	229	22.4±11.5	12.5±12.5	20.28±8.91	0.075
Urinary magnesium (mg/24h)	222	72.12±41.7	77.58±45.23	60.96±30.78	**0.0008**
FE Mg magnesium (%)	229	5.08±3.53	5.20±3.88	4.86±2.71	0.69

CRP: C reactive protein; UACR: urinary:albumin creatinine ratio; SBP: systolic blood pressure; ; FE: fractional excretion.

Table S3a. Multivariate model for prediction of rapid progression in women, adding UACR to the best multivariate model.

Variable	Coef.	OR	95% CI	P
Folic acid (pg/ml)	-0.192	0.826	(0.587-0.978)	0.121
SBP (mmHg)	0.078	1.082	(1.030-1.161)	**0.000**
UACR (mg/g)	0.001	1.001	(1.046-3.521)	0.242
FE Mg (%)	-0.478	0.620	(0.372-0.918)	**0.020**
Uric Acid (mg/dl)	0.607	1.835	(1.046-3.521)	**0.035**
Constant	-14.014			0.009

UACR: urinary:albumin creatinine ratio; SBP: systolic blood pressure; FE: fractional excretion.

Table S3b. Multivariate model for prediction of rapid progression in women replacing folic acid by UACR as a predictor.

**Variable**	**Coef.**	**OR**	**95% CI**	**P**
UACR (mg/g)	0.001	1.001	(1.000-1.003)	**0.016**
SBP (mmHg)	0.086	1.09	(1.029-1.150)	**<0.0001**
FE Mg (%)	-0.518	0.596	(0.352-0.890)	**0.009**
Uric Acid (mg/dl)	0.638	1.892	(1.098-3.568)	**0.022**
Constant	-16.96			0.002

UACR: urinary:albumin creatinine ratio; SBP: systolic blood pressure; FE: fractional excretion.

Supplementary figures:

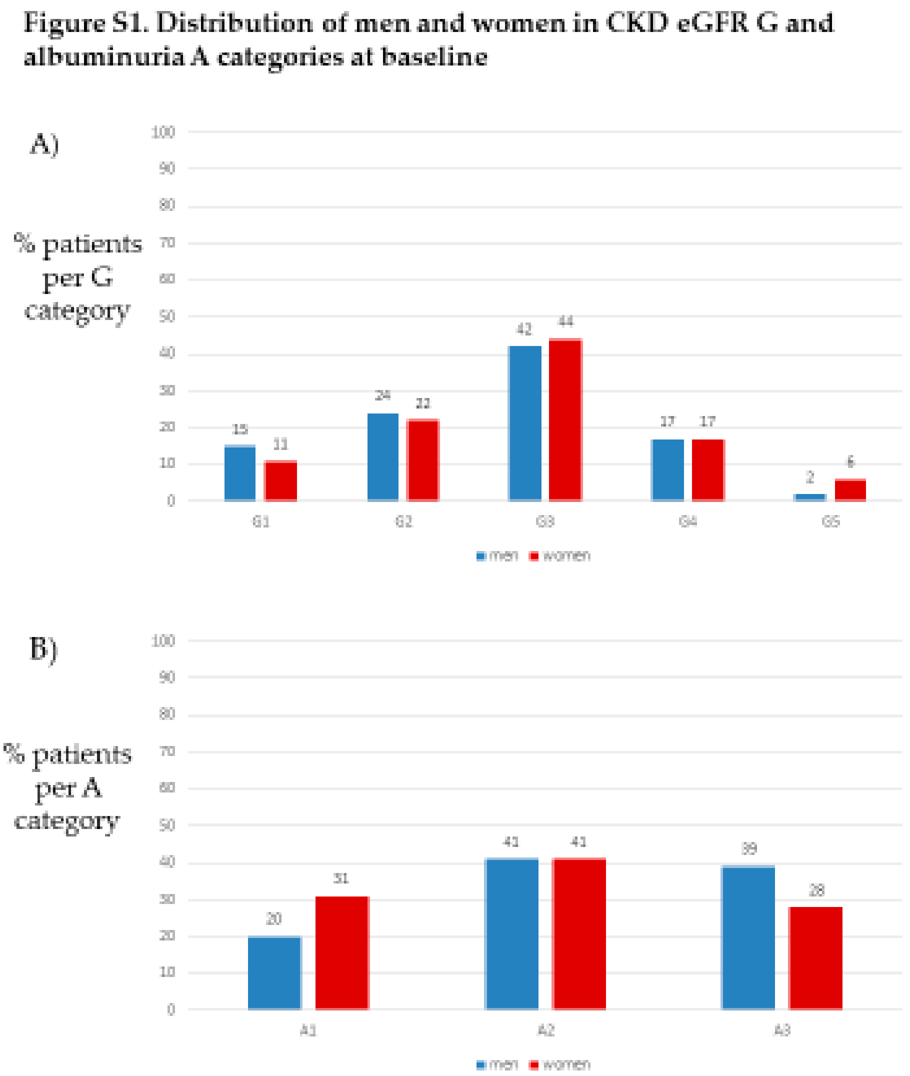

Figure S1. Distribution of men and women in CKD eGFR G and albuminuria A categories at baseline. A) Percentage of men and women per CKD eGFR G category. **B)** Percentage of men and women per CKD albuminuria A category. G and A categories defined as per KDIGO 2012. Percentages referred to the total number of men and the total number of women, respectively.

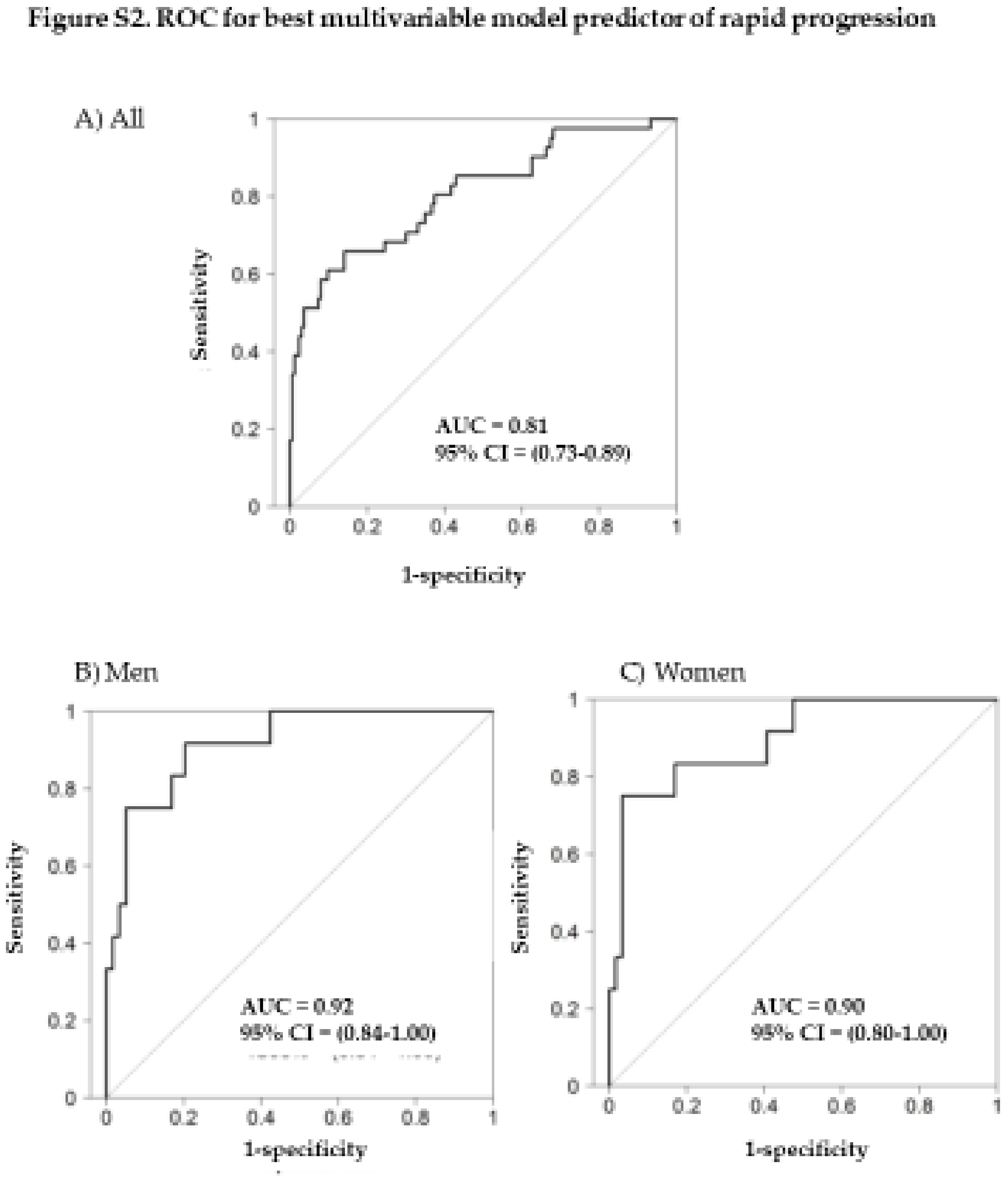

Figure S2. ROC for best baseline multivariable model predictor of rapid progression. Area under the curve (AUC) of the receiver operating characteristic (ROC) for prediction of rapid progression (defined as loss of eGFR >5 ml/min/1.73 m^2/year according to KDIGO 2012 by the best baseline multivariable model. **A)** All patients. **B)** Men. **C)** Women.
